# Lateral Hypothalamic Control of the Ventral Tegmental Area: Reward Evaluation and the Driving of Motivated Behavior

**DOI:** 10.3389/fnsys.2017.00050

**Published:** 2017-07-06

**Authors:** Susan M. Tyree, Luis de Lecea

**Affiliations:** Department of Psychiatry and Behavioral Sciences, Stanford UniversityStanford, CA, United States

**Keywords:** lateral hypothalamus, ventral tegmental area, motivated behavior, reward

## Abstract

The lateral hypothalamus (LH) plays an important role in many motivated behaviors, sleep-wake states, food intake, drug-seeking, energy balance, etc. It is also home to a heterogeneous population of neurons that express and co-express multiple neuropeptides including hypocretin (Hcrt), melanin-concentrating hormone (MCH), cocaine- and amphetamine-regulated transcript (CART) and neurotensin (NT). These neurons project widely throughout the brain to areas such as the locus coeruleus, the bed nucleus of the stria terminalis, the amygdala and the ventral tegmental area (VTA). Lateral hypothalamic projections to the VTA are believed to be important for driving behavior due to the involvement of dopaminergic reward circuitry. The purpose of this article is to review current knowledge regarding the lateral hypothalamic connections to the VTA and the role they play in driving these behaviors.

## Introduction

The field of behavioral neuroscience has taken a keen interest in understanding the neural circuits driving motivated behaviors. Food- and drug-seeking behaviors have received significant attention due to a concentrated effort to develop new, more successful treatments for disorders such as drug-abuse and obesity. Motivation is considered to be the energizing and directing of an animal’s behavior toward a specific reward or goal, giving the animal the energy or drive required to overcome the physical costs involved (i.e., climbing, fighting, hunting) as well as directing the animal’s concentration to the relevant activity over other possible activities (for example, feeding, drinking, lever pressing). It is vital that researchers work to develop a greater understanding of motivated behaviors due to their importance for survival; when an animal’s ability to successfully direct their energy toward important survival functions (such as eating, drinking and sleeping) is jeopardized, this can result in disordered states such as obesity/anorexia, drug addiction and sleep disruption.

A series of studies carried out by Anand and Brobeck (1951) brought attention to the lateral hypothalamus (LH) as a candidate neural structure involved in behavioral motivation. They showed that stimulating the lateral hypothalamic area resulted in increased food intake, and conversely, LH lesions caused aphagia and weight loss, which led the authors to label the LH a feeding center. Additionally, the LH projects densely to the ventral tegmental area (VTA), which is also known to play a role in not only food-reward, but reward in general and that these functions in the VTA rely on its population of dopaminergic neurons (Gallistel et al., 1985; Phillips et al., 2003; Grace et al., 2007) suggesting that these two structures are important for driving goal-oriented behaviors. Rather than reviewing the many brain structures involved in the behavioral motivation circuit (for review see Bailey et al., 2016; James and Aston-Jones, 2016), this review article will focus on the role of the LH and LH projections to the VTA in driving motivated behaviors.

## The Lateral Hypothalamic Structure and Function

The LH has been implicated in numerous functions including sleep-wake transitions (Adamantidis et al., 2007, 2010; Carter et al., 2009), feeding (Anand and Brobeck, 1951), energy balance (Brobeck, 1946), stress (Bonnavion et al., 2015) and reward (Olds and Milner, 1954; Hoebel and Teitelbaum, 1962) and plays a critical role in maintaining physiological and behavioral homeostasis. As well as being dubbed a “feeding center” by Anand and Brobeck (1951), the LH has also been labeled as a “pleasure center” (Olds, 1970) after it was shown that electrode implantation into the medial forebrain bundle in the LH resulted in persistent intracranial self-stimulation (ICSS; Olds and Olds, 1965). It has been suggested that this behavior is a result of stimulation of the descending fibers in the medial forebrain bundle that feed into the VTA (Bielajew and Shizgal, 1986), likely triggering a reward response.

The LH is a part of the hypothalamus located in the midbrain, and is home to a heterogeneous population of neurons. These populations include both gamma-aminobutyric acid (GABA)-ergic and glutamatergic neurons as well as subpopulations of neurons expressing neuropeptides that have been linked to the modulation of motivated behaviors such as hypocretin (Hcrt, also known as orexin; de Lecea et al., 1998; Sakurai et al., 1998), melanin-concentrating hormone (MCH; Qu et al., 1996), cocaine- and amphetamine-regulated transcript (CART; Kristensen et al., 1998), neurotensin (NT; Luttinger et al., 1982), leptin receptor (LepRb; Leinninger et al., 2011) and galanin (Skofitsch et al., 1985; Melander et al., 1986). These neurons connect to other brain structures via efferent projections from the LH to multiple structures including the amygdala, hippocampal formation, thalamus, the pons, brainstem and spinal cord, as well as intra-structural projections within the LH to other hypothalamic subnuclei (Ricardo and Koh, 1978; Berk and Finkelstein, 1982; Ter Horst and Luiten, 1987; Ter Horst et al., 1989). The LH also projects densely onto the VTA (Phillipson, 1979; Watabe-Uchida et al., 2012).

The large amount of overlapping gene expression within the LH is matched by the complexity of LH inputs into its target structures as well as feedback signals from those structures. Indeed, Horvath et al. (1999) showed images of a single neuron in the arcuate nucleus expressing immunoreactivity for neuropeptide Y, Hcrt inputs (from the LH) and a receptor for satiety hormone leptin. This suggests that the LH targets are equally heterogeneous as the LH itself. The large number of inputs, outputs, neuron types and functions present within the LH suggest that this structure is very complex and hosts an extremely diverse population of neurons, with many neurons co-expressing multiple neuropeptides and projecting to numerous target neural structures (for a comprehensive review of these connections, see Bonnavion et al., 2016), moving forward it will be important to gain a more precise understanding of these neurons, projections and functions to better disentangle the many roles of the LH.

## The Ventral Tegmental Area Structure and Reward Function

The VTA is a semi-circular nucleus which lies along the midline in the midbrain, it is home to a heterogeneous population of neurons containing multiple neurotransmitters including NT (Kalivas and Miller, 1984), cholecystokinin (CCK; Studler et al., 1981) and dopamine (for a thorough neuroanatomical review, see Oades and Halliday, 1987). The VTA dopaminergic system in particular has been implicated in brain-stimulation reward and food reward, psychomotor stimulation, learning and memory formation (Yokel and Wise, 1975; De Wit and Wise, 1977; Berridge, 2007; Friedman et al., 2014; Popescu et al., 2016), and it has been shown that goal-directed behavior is promoted by dopamine release from VTA^DA^ neurons (Gallistel et al., 1985; Phillips et al., 2003; Grace et al., 2007). Studies have shown that both the synaptic connections and intrinsic excitability of DA neurons are highly plastic dependent on the experiences of the animals (Stuber et al., 2008; Mao et al., 2011; Collo et al., 2014; Friedman et al., 2014; Gore et al., 2014). This suggests the possibility for experience/outcome-based modulation of behavioral motivation to be mediated via VTA^DA^ neurons and a “directing” role for dopamine in goal-oriented behaviors.

VTA^DA^ neuron involvement in reward processing has been studied extensively in an attempt to understand how these neurons code for rewards and the mechanisms through which they are able to modulate animal behaviors. However the complexity of the VTA, as well as the LH inputs into the VTA, require equally complex methods to investigate specific neuron populations within such heterogeneous neuron populations. Notably Eshel et al. (2015) carried out a complex set of experiments using a multi-method approach combining computational modeling, extra-cellular recordings, optogenetics and viral injections to investigate the computational mechanisms by which VTA^DA^ neurons calculate reward prediction error. Performing extra-cellular recordings of DA neurons while delivering expected and unexpected rewards, and using subsequent optogenetic manipulations to investigate the importance of VTA^GABA^ neurons to normal VTA^DA^ function. They found that as the size of the reward the animal receives increases, so does the DA neuron response, which was consistent with previous results (Tobler et al., 2005; Cohen et al., 2012), they also found that expectation of a reward resulted in a suppression of the DA neuron response, and that this response fit to a subtractive computational model better than an alternative divisive model (Eshel et al., 2015). Then, Eshel et al. (2015) investigated the role of VTA^GABA^ neurons in their subtraction model of VTA^DA^ neuron suppression in expected rewards by optogenetically mimicking normal VTA^GABA^ neuron firing patterns and observing VTA^DA^ activity. They found that VTA^GABA^ stimulation resulted in the suppression of DA responses to unexpected rewards in a similar pattern to that seen in animals receiving expected rewards. This VTA^GABA^-induced suppression of DA responses also fit with a subtractive computational model. Additionally, they showed that inhibition of VTA^GABA^ neurons partially reversed the expectation-dependent suppression of VTA^DA^ reward responses. Taken together this suggests that VTA^DA^ neurons calculate reward-error using a subtractive model, and that VTA^GABA^ neurons play a role in the temporal expectation modulation of DA responses in a manner that is consistent with the ramping expectation function in some models of prediction error computational models (Hazy et al., 2010; Rivest et al., 2014). This modulation of reward response in the VTA may play an important role in directing motivated behaviors to rewards that are less predictable over rewards that are more regularly available. This also suggests a mechanism by which VTA^DA^ neurons can rationalize between multiple rewards within an environment by modulating the reward value of more reliable rewards to be less rewarding than unpredictable rewards to shift the animals drive to focus on less readily-available rewards. This series of experiments shows the multitude of benefits that can be gained by using a multi-method approach to investigate neural circuits, by using behavioral protocols, optogenetics, electrophysiology and computational modeling (as well as investigating the role of both DA and GABA activity for comparison) these researchers were able to gain a deeper understanding of VTA^DA^ activity by observing it from multiple angles.

## The LH → VTA Circuit Connections Linked to Motivated Behaviors

Connections between the LH and the VTA have been studied extensively regarding their role in motivation, particularly LH inputs into the VTA dopaminergic system. Both DA depletion and excitotoxic LH lesions have similar outcomes altering motivated behavior, including aphagia (Grossman et al., 1978; Stricker et al., 1978). Shizgal et al. (1980) showed that the majority of reward-relevant fibers in the LH (identified using a self-stimulation protocol) project toward the VTA showing a clear neurophysiological connection between these two structures relating to reward. Early electrophysiological studies in the LH showed that LH stimulation could trigger a variety of behaviors such as mating, feeding, drinking, nest-building and gnawing (Roberts and Carey, 1965; Caggiula and Hoebel, 1966; Mogenson and Stevenson, 1967). Interestingly, these different behaviors did not appear to correlate to topographically distinct stimulation regions within the LH (Wise, 1971) but instead to patterns that had developed over a number of trials according to what type of goal stimuli the animals were presented with Valenstein et al. (1968), Wise (1968). This suggests that perhaps this LH stimulation was initiating a more general drive response rather than initiating a specific goal-targeted behavior. Considering the role of VTA in calculating prediction errors and this could mean that the LH triggers the “drive” component of motivation and the VTA is playing the role of “directing” that motivation toward goals within the animal’s environment, changing the focus of the motivation as the rewards within the environment change.

The development of new techniques and biomarkers has allowed a closer look at the roles of different populations of LH neurons in VTA function. For example, optogenetic stimulation of LH^GABA^ inputs to the VTA results in conditioned place preference (Barbano et al., 2016), reduces VTA^GABA^ activity, and drives nucleus accumbens dopamine release (Nieh et al., 2016). Nieh et al. (2016) additionally showed that optogenetic stimulation of glutamatergic LH inputs to the VTA results in conditioned place aversion. Interestingly, the behavioral response to optogenetic stimulation of VTA-innervating LH^GABA^ neurons differed depending on the stimulation frequency, with low frequency stimulation (5–10 Hz) resulting in increased feeding, and high frequency stimulation (40 Hz) appeared to trigger reward, resulting in a place preference (Barbano et al., 2016). It is possible that these two functions may be being mediated by two different neuropeptides co-expressed within LH^GABA^ neurons, or that this stimulation triggers a general “drive” and the stimulation frequencies result in the release of different neuropeptides in the VTA, resulting in a target for the “drive” response. This could suggest that the LH and the VTA are the “drive” and “focus” sources for motivated behaviors, respectively, with LH activation producing a general energizing of the animal to perform a behavior, and the VTA then directing that energy to a specific goal-oriented behavior—depending on which neurotransmitters are released, or the stimulation frequency, or some other determining factor. Additionally, the development of this gene-targeting methodology also opens up the possibility to investigate the multiple other neuron types in the LH that are known to project to the VTA to better understand how these neuron types differentiate between multiple input signals and determine which environmental goals to pursue.

### A Role for Hypocretin in Motivation and Reward

Studies investigating connections between the LH and the VTA have also investigated whether there are specific populations of LH neurons underlying the connection with the VTA. One LH → VTA population of interest is Hcrt neurons, which are known to project densely to the VTA (Peyron et al., 1998; Fadel and Deutch, 2002) and are suggested to play an important role in the LH → VTA^DA^ reward circuit. Within the LH Hcrt-containing neurons (Hcrt-1 and Hcrt-2 also referred to as ORX-A and ORX-B, respectively), are concentrated particularly in the perifornical of the LH, and are known to project widely throughout the brain (de Lecea et al., 1998; Peyron et al., 1998; Sakurai et al., 1998, 2005; Marcus et al., 2001; Fadel and Deutch, 2002; Yoshida et al., 2006) and produce effects via actions at their receptors HcrtR1 and HcrtR2 (de Lecea et al., 1998; Peyron et al., 1998; Sakurai et al., 1998; Marcus et al., 2001; Fadel and Deutch, 2002). Hcrt has been shown to play a prominent role in sleep-wake transitions (de Lecea et al., 1998; Hagan et al., 1999; Piper et al., 2000; España et al., 2001; Adamantidis et al., 2007, 2010; Carter et al., 2009) as well as reward/reinforcement (Boutrel et al., 2005; Harris et al., 2005; Smith et al., 2010) and previous studies have shown that Hcrt^LH^ neurons have modulatory effects on VTA function (Harris et al., 2005; Borgland et al., 2006).

Pharmacological and genetic methods of inducing or inhibiting Hcrt activity have provided evidence that Hcrt is involved in DA reward processes particularly via connections with the VTA (España et al., 2010, 2011; Prince et al., 2014). There is also evidence that Hcrt directly affects dopamine activity and reward responses; it has been shown that Hcrt administration induces burst firing of DA neurons (Borgland et al., 2006) and results in increased cocaine self-administration (España et al., 2011). Chemical Hcrt activation and Hcrt infusions into the VTA reinstate previously extinguished drug- and food-seeking behaviors (Harris et al., 2005). Hcrt has also been shown to be necessary for normal behavioral and neural reward responses, Hcrt knock-out mice fail to develop a cocaine-conditioned place-preference, and showed diminished DA signaling following cocaine administration compared to wild-type controls (Shaw et al., 2016). Administration of Hcrt antagonist almorexant in the VTA attenuates ethanol self-administration (Srinivasan et al., 2012), and blocking Hcrtr1 using SB-334867 results in reduced excitation of DA neurons in the VTA (Moorman and Aston-Jones, 2010) and decreases motivation to obtain cocaine rewards (Borgland et al., 2006; España et al., 2010; Prince et al., 2014; Brodnik et al., 2015). Taken together these studies suggest that intact Hcrt function is necessary for both neural dopaminergic responses and behavioral responses to reward and reinforcement.

Considering the various roles for Hcrt in behavioral arousal, LH^Hcrt^ may initiate a general drive response, and the target behaviors of this increased drive may be determined in the targets of Hcrt neurons. Based on the evidence for Hcrt modulation of VTA^DA^ activity, it would appear that there is some link between LH^HCRT^ and VTA^DA^ neurons, however, studies investigating connections between these two neuron populations have discovered that this LH^HCRT^ modulation of VTA^DA^ neurons does not appear to be driven by a straightforward monosynaptic connection. It has been shown that few Hcrt axons in the VTA synapse directly onto VTA^DA^ or VTA^GABA^ neurons and the majority of Hcrt fibers appear to be passing fibers, possibly passing on to caudal brainstem structures (Balcita-Pedicino and Sesack, 2007). This suggests that these Hcrt → VTA inputs may be playing a modulatory role in the VTA via non-synaptic mechanisms or volume transmission, rather than direct monosynaptic inputs. Although the majority of reward-relevant fibers in the LH do project toward the VTA (Bielajew and Shizgal, 1986) and infusion of Hcrt-2 in VTA whole-cell patch-clamp recordings increases glutamatergic transmission to VTA neurons (Borgland et al., 2008), there have been findings which raise questions about the mechanisms of LH → VTA circuitry in mediating reward: paired-pulse studies of LH → VTA fibers have shown that both the refractory periods for fibers depolarized at the electrode tip and their conduction velocities are significantly faster than would be expected from the unmyelinated dopaminergic fibers connecting the VTA and LH (Yeomans, 1979; Shizgal et al., 1980; Gallistel et al., 1981). The finding that the timescales of these fibers do not line up suggest that the mechanism via which LH neurons mediate VTA^DA^ neurons may be more complex than previously considered and requires further investigation. These discrepancies can be further studied to develop models to determine possible mechanisms that would explain the divergent timescales. Tools such as optogenetics will be particularly useful for this purpose due to the rapid induction of neuronal activity and ability to manipulate neuronal activity with timed precision, this method has been used to investigate timescales of LH^Hcrt^ connections to the locus coeruleus and develop a network model accounting for variations in neuronal activity that have been observed in slice electrophysiology (Mosqueiro et al., 2014) this method could also be applied to the LH^Hcrt^ → VTA^DA^ circuit. It has previously been shown that Hcrt neurons take 30 s to peak in slice electrophysiology recordings (Ishibashi et al., 2015) understanding how this fits into a model of Hcrt interactions with the VTA will be important for determining mechanisms underlying this Hcrt modulation of DA signaling.

### Other LH Neuropeptide Candidates Involved in Motivation and Reward

Timing discrepancies between Hcrt and DA signaling could also be due to the involvement of additional LH neurotransmitters that have been linked to driving motivated behaviors. A recent study highlighted CART as a candidate in the LH → VTA^DA^ circuit using ICSS with an electrode implanted in the LH. Somalwar et al. (2017) showed that ICSS resulted in increased activation of CART cells in the LH and that administration of CART into the posterior VTA enhanced the self-stimulation behavior. Additionally, they showed that animals avidly self-infused CART (55–102) into the posterior VTA via cannula, and that this behavior can be inhibited by administration of dopamine D1 receptor antagonist directly into the nucleus accumbens shell. Suggesting that CART may too play a role in VTA^DA^ reward processes. Another possible candidate is NT, which is known to project from the LH to the VTA (Leinninger et al., 2011) and has been shown to facilitate prolonged DA release (Patterson et al., 2015). It has also been shown that LH NT neurons synapse onto twice as many VTA^DA^ neurons as they do VTA^GABA^ neurons (Beier et al., 2015). The LH is home to many different neuropeptides and receptor types, and many LH neurons co-express multiple genes (for a comprehensive review see Bonnavion et al., 2016). For example, approximately 30% of LH NT neurons co-express the LepRb receptor for anorexigenic hormone leptin (Leinninger et al., 2011), and 95% of these neurons co-expressing NT and LepRb also co-localize with galanin (Laque et al., 2013).

## Future Investigations of The LH → VTA Circuit: What Can We Learn?

**Figure 1:**
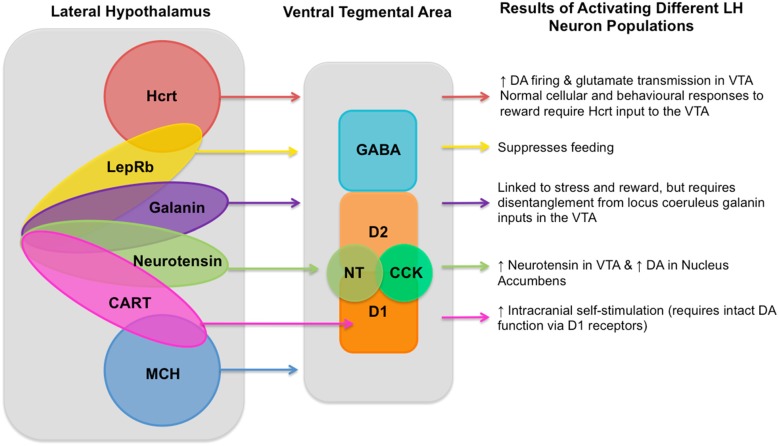
Known lateral hypothalamus (LH) neuronal population inputs into the ventral tegmental area (VTA) and the functions linked to their activation. A diagram showing neuron populations in the LH, their currently known inputs into the VTA, and the behavioral and neural responses to their activation as described in the article text. LH cell types include: hypocretin (Hcrt; de Lecea et al., 1998; Sakurai et al., 1998) known to produce increased dopaminergic firing (Borgland et al., 2006) and glutamate transmission in the VTA (Borgland et al., 2008); leptin receptor (LepRb; Leinninger et al., 2011) which is involved in processing dietary fat intake and satiety (Leinninger and Myers, 2008; Vong et al., 2011); galanin (Skofitsch et al., 1985; Melander et al., 1986) which is linked to stress and reward (Picciotto et al., 2010), but requires disentangling from locus coeruleus galanin inputs to the into the VTA; neurotensin (NT; Luttinger et al., 1982) which produces increased NT in the VTA and subsequently increased dopamine in the Nucleus Accumbens (Patterson et al., 2015); cocaine- and amphetamine-regulated transcript (CART; Kristensen et al., 1998) which triggers increased intracranial self-stimulation (ICSS) which requires intact DA function via the D1 receptor (Somalwar et al., 2017); and melanin-concentrating hormone (MCH; Qu et al., 1996). As well as these LH neuron types there are also gamma-aminobutyric acid (GABA)-ergic and glutamatergic neurons, though the degree to which each of these different neuron types in the LH can be determined to be glutamatergic or GABAergic still requires further research and so is not represented in this diagram. VTA neuron types include GABAergic neurons, two types of dopaminergic neurons (D1 and D2), as well as NT (Kalivas and Miller, 1984) and cholecystokinin (CCK; Studler et al., 1981). Within the LH it is known that single neurons can co-express multiple different neurotransmitters, known overlapping populations are shown above, though the size of the overlapping circles is not intended to be representative of the size of the overlapping neuron populations and should be merely considered as illustrative.

While the broad connectivity between LH and VTA has been clearly elucidated, disentangling the precise roles of the relatively heterogeneous subpopulations of LH → VTA circuit neurons has made slow progress due to the topological and functional overlap of these LH neuronal populations, their various functions (illustrated in Figure 1), their diverse connections to the VTA, as well as feedback signals traveling from the VTA to the LH. These factors make investigating this circuit difficult, however the recent development of new technologies has provided researchers with more precise tools to investigate this circuit that should be taken advantage of.

Untangling the complexity of neurons co-expressing multiple genes can be investigated with gene-targeted methods such as optogenetics and fiber-photometry, methods which have been successfully used to investigate other LH circuits such as the LH-LC arousal circuit (Adamantidis et al., 2010). These methods could be used to investigate the role of each of these LH neuron populations in driving VTA^DA^ function, whether the subpopulations of NT or CCK neurons in the VTA are playing an important role in LH mediated VTA activity. Of particular interest will be the investigation of how LH^Hcrt^ neurons drive VTA^DA^ neurons, as it is apparent that there is some modulation in this direction, however the lack of direct LH^Hcrt^ → VTA^DA^ connections raises questions as to the mechanism behind this modulatory function. Additionally these methods could be used to study how LH inputs into the VTA and VTA inputs into the LH modulate this neural circuit. Researchers can also benefit from using multi-method approaches, such as those reported by Eshel et al. (2015) to develop a more nuanced understanding of the multi-faceted functions of the LH and VTA.

We have already seen how the transition from gross electrophysiological stimulation and recording to precise and neuron-type specific optogenetic stimulation and fiber-photometry recordings has allowed researchers to genetically target (rather than topographically target) much more specific and well defined neuron populations than the prior large-scale neuron-type agnostic electrophysiological and pharmacological approaches. Additionally, the rapid development of next-generation sequencing approaches allows researchers to investigate heretofore unknown or indefinable single-cell gene-expression profiles (Macosko et al., 2015). These approaches are key in the identification of the different neuron types within a topologically heterogeneous and complex structure such as the LH, and in combination with optogenetics and fiber photometry will allow fine-grained dissection of the role of specific neuron populations in ways that were not previously possible. Indeed, a recently developed method for profiling cell-types by separating cells into Nano liter-sized droplets and sequencing each cells RNA (drop-seq) was recently used to identify 50 distinct cell types within the hypothalamic arcuate-median eminence complex (Campbell et al., 2017). A similar experiment in the LH will be crucial to give researchers the ability to cluster neurons into populations based on their gene expression. This could lead to a better categorization of different cell types within the diverse population of LH neurons and subsequent optogenetic investigations targeting these genetically distinct neuron populations could lead to a better understanding of the roles these neuron subpopulations play in functions such as motivating behavior.

## Conclusion

The LH → VTA circuit clearly plays an important role in driving behavior. Initial studies showed the importance of the LH in motivating basic functions such as mating, feeding, drinking, nest-building and gnawing, and the evidence that lesioning the LH results in a loss of these behaviors such as dramatic weight-loss, has highlighted the importance of this circuit for survival. Understanding this circuit will be important for understanding how normal behavior is elicited, and what is going wrong when these behaviors become disordered in cases such as obesity, anorexia, drug-abuse, anhedonia, etc. Considering the two main facets of goal-oriented behavior are the energizing and directing of behavior and what is known of the LH and VTA, it would appear that the LH neurons may play more of a role in the “driving” motivated behavior whereas the VTA likely directs the behavior toward relevant goals/rewards via the dopaminergic system for example, by modulating the reward-value of different environmental rewards. Although there are still many outstanding questions regarding the LH → VTA circuit, the development of new research technologies are allowing researchers more promising opportunities to probe this circuit and gain a more specific understanding of the basis for the dysregulation of this circuit and the negative behavioral consequences associated with it, such as drug abuse and obesity.

## Author Contributions

This article was written by SMT with editorial help and guidance from LL.

## Conflict of Interest Statement

The authors declare that the research was conducted in the absence of any commercial or financial relationships that could be construed as a potential conflict of interest.
